# Vestibular evoked myogenic potential: new perspectives in multiple sclerosis

**DOI:** 10.1016/S1808-8694(15)31284-2

**Published:** 2015-10-20

**Authors:** Renata Chade Aidar, Fábio A. Suzuki

**Affiliations:** 1 Master in Otorhinolaryngology – IAMSPE – SP. Instituto de Assistência Médica ao Servidor Estadual “Francisco Morato de Oliveira”; 2 Ph.D., Assistant Physician, Vice-coordinator of Post-graduation, IAMSPE, Instituto de Assistência Médica ao Servidor Estadual “Francisco Morato de Oliveira”

**Keywords:** auditory evoked potentials, vestibular function exam, multiple sclerosis/diagnosis

## Abstract

**Summary:**

**Aim**: To evaluate vestibular evoked myogenic potentials in patients with multiple sclerosis as method of diagnostic support.

**Study design:**

Case-control.

**Material and Method:**

We studied a group of normal individuals (n = 15) and a Studied group (n = 15) that comprised patients with diagnosis of multiple sclerosis. Both groups were submitted to vestibular evoked myogenic potential exam. In each ear it was applied 200 stimuli by clicks and repeated for 2 consecutive cycles with the purpose of evaluating reproducibility. The active electrode of surface was put on the superior S‡of sternocleidomastoid muscle and the reference electrode on the anterior border of the clavicle. The individuals were instructed to rotate theirs head to the opposite side to the stimulated ear.

**Results:**

Vestibular evoked myogenic potential responses were prompt, reproducible and biphasic. The latency of wave P1 and N2 and P1-N2 amplitude showed a higher value in the studied group when compared with the normal group. There was no significant difference when the ears were compared in P1 and N2 amplitude. We noticed that individuals with multiple sclerosis showed no response in 30% of the cases. In evaluating the individuals of the Studied group with otoneurology symptoms and compared with individuals without symptoms, it was observed that P1 and N2 latencies and P1-N2 amplitude were higher in symptomatic cases.

**Conclusion:**

Vestibular evoked myogenic potential was considered a good method of diagnostic support of vestibulospinal tract in cases of multiple sclerosis.

## INTRODUCTION

Vestibular evoked myogenic potentials (VEMP) is a test that assesses vestibulospinal pathway from the sacculus macula. VEMP acoustic stimulus is evoked by clicks presented through headsets. The macula is activated by sound stimulus, generating an electrical potential that goes through the pathway of the inferior vestibular nerve, lateral vestibular nerve, vestibulospinal tract and finally, ipsilateral motor neuron of neck muscle^1^. Vestibular nuclei are located in the pons, placed on the floor of the IV ventricle, and they are divided into: medial, inferior, lateral and superior. Vestibulospinal tract has descending fibers from the lateral vestibular nucleus.

Some authors studied evoked myogenic responses with active surface electrode placed on the inion and stated that the response was mediated by the vestibular system^2^, ^3^. As time went by, studies started to place surface electrodes on the sternocleidomastoid muscle^4^, ^5^, ^6^, ^7^, ^8^, ^9^, ^10^, ^11^, ^12^, ^13^, ^14^, ^15^, ^16^, ^17^.

According to articles published in the literature, VEMP is a test that assesses brainstem damage or lesions that affect vestibulospinal tract, such as the case of multiple sclerosis (MS)^1^, ^18^. MS is an inflammatory demyelinizing disease of central nervous system (CNS) of autoimmune etiology. However, it is a disease with multifactorial etiology, in which the association of genetic predisposition and external factors would be determining for triggering immune events related to inflammatory and demyelinizing process of CNS. Epidemiological studies showed heterogeneous distribution of the disease in the world and incidence of 0.1% in countries of moderate temperature. In tropical countries, such as Brazil, the disease is rare, but recent data reported high incidence in Caucasian and African-Brazilian subjects^19^.

As to diagnosis, there is evidence of two or more lesions in CNS white matter in imaging exams, preferably within one month of interval between symptoms, in patients aged 15 to 50 years. Many patients experience clinical recovery from acute episodes and progress to remission stages. Lesions in the CNS white matter may not be manifested by symptoms and therefore, they may be diagnosed only through specific diagnostic tests^20^.

In the past, the diagnosis of MS was based on clinical data, but as a result of technology advance, there has been an increase in early diagnosis. The most used tests are magnetic resonance imaging (MRI), evoked potentials (EP), and analysis of immunoglobulin in cerebrospinal fluid (CSF). MRI and EP provide information on symptomatic and asymptomatic stages, and on atypical characteristics. CSF analysis reveals information on the inflammatory process. Neuroimaging, through head and spinal cord MRI, is particularly important in the diagnosis and progression of the disease. Good clinical history, physical examination and clinical evolution are essential to understand the disease, as shown by Poser and McDonald criteria^21^, ^22^. Patients with MS present different otoneurological symptoms. The most common complaint is dizziness, more specifically imbalance. The next most common complaints are ear fullness, tinnitus and vertigo^22^.

According to the above-reported, EP are indicated for diagnosis, meaning that VEMP could be an additional diagnostic method for investigation in cases of MS. Therefore, we decided to apply VEMP in subjects with diagnosis of MS. It is an objective, non-invasive and easy to perform test. It is not uncomfortable and patients do not have to make physical efforts.

## OBJECTIVES

1. To assess responses to VEMP in patients with MS and compare them to the control group.
2. To correlate otoneurological complaints with results of latency and amplitudes in VEMP.
3. To demonstrate that VEMP is a good method for the assessment of vestibulospinal tract in patients with MS.

## MATERIAL AND METHOD

VEMP study started with the control group comprising 15 subjects. The division of Neurology, IAMSPE, referred to the division of Otorhinolaryngology, 15 patients with diagnosis of MS. To diagnose MS, we relied on the criteria set forth by Poser and McDonald^21^. Both criteria systems are based on number of crises, high resolution computed tomography (HRCT) and CSF and EP affections. The second system is complemented by MRI findings.

The inclusion criteria were: subjects in the control group without otoneurological complaints and in the studied group, subjects with confirmed diagnosis of MS. The exclusion criteria for both groups were: malformation of pinna and external auditory canal and limitation of neck rotation movement.

Before VEMP, patients with MS and subjects in the control group were submitted to clinical history and ENT physical examination. To perform VEMP, we used device Nihon Khoden programmed to middle-latency evoked potential. Stimuli were sent through headsets brand Elega type DR 531.

**Figure 1:**
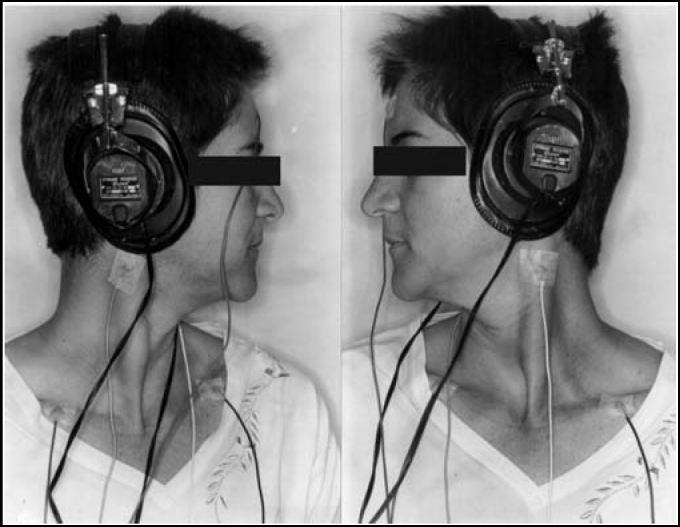
Position of the patient in lateral rotation and position of reference and ground surface electrodes

After skin cleaning with the appropriate material, surface electrodes were placed on the following positions: active, on the upper 1/3 of sternocleidomastoid muscle, reference electrode, on the anterior margin of ipsilateral clavicle, and ground electrode, on the forehead. A small amount of electrolytic material was applied to the surface of the electrode and fixed with adhesive tape (Figure 1). Patients were instructed to sit down on a chair and rotate the head contralaterally to the stimulated ear. The contraction of muscle was maintained thanks to the cooperation of patients in maintaining the same position during the test.

To perform VEMP we used sound stimuli presented as rarefaction clicks of 0.1ms of duration with 5ms/div and frequency of 2Hz; 200 stimuli were applied to each ear and repeated three consecutive times at 95 dB HL. All responses were filtered between 20 and 1000 Hz.

VEMP response was obtained after monoaural stimulation, including masking applied to stimulus intensity minus 40 dB HL. VEMP tracings were biphasic positive-negative P1-N2 waves.

To analyze data, we used the following statistical methods: t-independent; McNemar test; chi-square test, t-paired test. The level of significance was 5% (p < 0.5).

## RESULTS

The mean age of control group was 32.8 years, with SD of 7.18 years (n = 15). The Studied group presented mean of 39.3 years with SD of 11.9 years (n = 15). There was no statistically significant difference between the groups (Table 1).

**Table 1:** Distribution according to age in years in the control group and studied group.

Statistics	Control group	Studied group
Mean	32.8	39.3
Standard deviation	7.18	11.9
Total	15	15

t-dependent test

p = 0.079

As to absence or presence of responses in the studied group comparing ear side, we found 26.7% of absence of right ear responses against 33.3% on the left; 73.3% of presence of right side responses against 66.7% on the left (Chart 1). There were no statistically significant differences between the ears.

**Chart 1:** Distribution concerning absence and presence of responses in patients with MS comparing both ears (N = 15).

Left side				
		Absent	Present	Total
Right side	Absent	01 (6.7%)	04 (26.7%)	05 (33.3%)
	Presente	03 (20%)	07 (46.7%)	10 (66.7%)
	Total	04 (26.7%)	11 (73.3%)	15 (100%)

Test: Mc Nemar

In Chart 2, we observed distribution of presence and absence of symptoms comparing the ears in the studied group. There was no statistically significant difference between the ears.

**Chart 2:** Distribution concerning presence and absence of symptoms in patients with MS comparing both ears.

Right side				
		Absent	Present	Total
Left side	Absent	02 (13.3%)	01 (06.7%)	03 (20.0%)
	Present	02 (13.3%)	10 (66.7%)	12 (80.0%)
	Total	04 (26.7%)	11 (73.3%)	15 (100%)

Test: Mc Nemar P = 1.000

Upon comparing the results of VEMP between the groups, we observed that 30% of the studied group presented absence of responses (Table 2). Therefore, we found statistically significant value (observed value = 10.59).

**Table 2:** Distribution concerning absence and presence of VEMP responses comparing the control group and MS group.

Wave	Control Group	Studied Group	Total
Absent	00 (0%)	09 (30.0%)	09 (15.0%)
Present	30 (100.0%)	21 (70.0%)	51 (85.0%)
Total	30 (100.0%)	30 (100.0%)	60 (100.0%)

Test: Chi-square

Observed value: 10.59*

Critical value: 3.84

Chi-square test assessed presence and absence of symptoms between the groups by comparing them with VEMP results (Table 3). There was no statistically significant difference. Even though non-significant, the group with presence of symptoms presented higher number of absent responses (34.8%), indicating a trend that could be better assessed with a larger studied group.

**Table 3:** Distribution concerning absence and presence of symptoms in the group with MS compared to VEMP responses.

	Otoneurological Symptoms		
	+	-	Total
Wave	N (%)	N (%)	N (%)
Absent	08 (34.8%)	01 (14.3%)	09 (30.0%)
Present	15 (65.2%)	06 (85.7%)	21 (70.0%)
Total	23 (100.0%)	07 (100.0%)	30 (100.0%)

Test: Chi-square

Observed value: 1.07

Critical value: 3.84

Using t-paired test, we analyzed the statistics of amplitude P1-N2, wave P1 and wave N2 in VEMP in the control group and studied group, comparing the right ear with the left ear. There was no statistically significant difference in all studied cases (Table 4, Table 5, Table 6, Table 7, Table 8, Table 9).

**Table 4:** Amplitude of wave PIN2 (μV) of the control group comparing right and left ears.

Statistics	Right side	Left side
Mean	7.54	7.23
Standard deviation	5.95	4.76
Total	15	15

T-paired test

p = 0.796

**Table 5:** Latency of wave P1 (ms) in VEMP in the control group comparing right and left ear.

Statistics	Right side	Left side
Mean	11.12	10.76
Standard deviation	1.64	1.59
Tamanho	15	15

T-paired test

p = 0.344

**Table 6:** Latency of wave N2 (ms) in VEMP in the control group comparing right and left ears.

Statistics	Right side	Left side
Mean	15.15	14.86
Standard deviation	2.51	2.85
Total	15	15

T-paired test

p = 0.613

**Table 7:** Amplitude of wave PIN2 (μV) in the MS group comparing right and left ears.

Statistics	Right side	Left side
Mean	12.17	13.30
Standard deviation	6.50	3.63
Total	7	7

T-paired test

p = 0.759

**Table 8:** Latency of wave P1 (ms) in the MS group comparing right and left ears.

Statistics	Right side	Left side
Mean	13.40	13.49
Standard deviation	2.87	1.77
Total	7	7

T-paired test

p = 0.944

**Table 9:** Latency of wave N2 (ms) in the MS group comparing right and left ears.

Statistics	Right side	Left side
Mean	18.64	17.84
Standard deviation	2.67	1.27
Total	7	7

T-paired test

p = 0.514

The control group was compared to the studied group by assessing the statistics of amplitude of P1-N2, wave P1 and wave N2 applying t-independent test. In the three studied situations, the studied group presented higher value in VEMP responses than the control group, which was considered statistically significant (Tables 10, 11 and 12).

**Table 10:** Amplitude P1N2 (μV) comparing control group and MS.

Statistics	Control	Studied
Mean	7.39	10.94
Standard deviation	5.30	5.39
Total	30	21

T-independent test

p = 0.023

**Table 11:** Latency of wave P1 (ms) comparing control group and MS group.

Statistics	Control	Studied
Mean	10.94	12.78
Standard deviation	1.60	2.41
Total	30	21

T-independent test

p = 0.005*

**Table 12:** Latency of wave N2 (ms) comparing the control group and MS group.

Statistics	Control	Studied
Mean	15.00	17.07
Standard deviation	2.64	2.73
Total	30	21

T-independent test

p = 0.009*

We assessed mean value of amplitude P1-N2, wave P1 and N2 in subjects with presence of symptoms and compared the group of patients with absence of symptoms.

In the three situations, the group of subjects with symptoms presented mean value of VEMP response higher than the group without symptoms (Tables 13, 14 and 15).

**Table 13:** Amplitude P1N2 (μV) comparing group and presence or absence of symptoms.

Statistics	+	-
Mean	12.37	7.34
Standard deviation	5.56	2.79
Total	15	6

T-independent test

p = 0.013*

**Table 14:** Latency of wave P1 (ms) comparing the group with presence and absence of symptoms.

Statistics	+	-
Mean	13.49	11.02
Standard deviation	2.24	1.99
Total	15	6

T-independent test

p = 0.030*

**Table 15:** Latency of wave N2 (ms) comparing the group with presence and absence of symptoms.

Statistics	+	-
Mean	18.05	14.63
Standard deviation	2.26	2.34
Total	15	6

T-independent test

p = 0.006*

## DISCUSSION

In the study described here, all subjects presented diagnosis of MS, defined by the division of clinical neurology, IAMSPE, based on the criteria by Poser and McDonald^21^, ^22^. In 1868, Charcot described the first set of diagnostic criteria for MS. Other criteria were published in 1954, Allison-Millar; 1961, Schumaker; 1972, McAlpine; finally, in 1983, Poser, and 2002, McDonald. The first criteria were based on subjective assessment of the researcher. The two last criteria systems were based on clinical impressions, laboratory analyses, neuroimaging and evoked potentials. However, to define the diagnosis it is necessary to associate the clinical history with physical examination and disease progression.^21^

When observing a study of middle latency potential, we observe responses within a period of 12 to 50 ms. In order to study the middle latency, we used VEMP in the following parameters: surface electrodes were placed on sternocleidomastoid muscle because responses are more consistent and homogenous, in addition to being a more practical and comfortable method to the patients^3^, ^4^, ^5^, ^6^, ^7^, ^8^, rarefaction clicks, frequency of 2Hz and pass filter that ranged from 20 to 1000Hz. There is variation of frequency range described in the literature. However, lowest frequency equal or below 5Hz is the best and most widely used by the studied literature^4^, ^6^, ^7^, ^8^, ^10^, ^12^, ^13^, ^15^, ^17^, ^23^.

Reproducibility is an important factor when conducting evoked potential test and it is extremely valuable both immediately and in the long-run. The immediate form confirms the existence of integrity of vestibulospinal tract and the long-run form evidences disease progression and its prognosis^8^, ^11^, ^24^.

**Figure 2:**
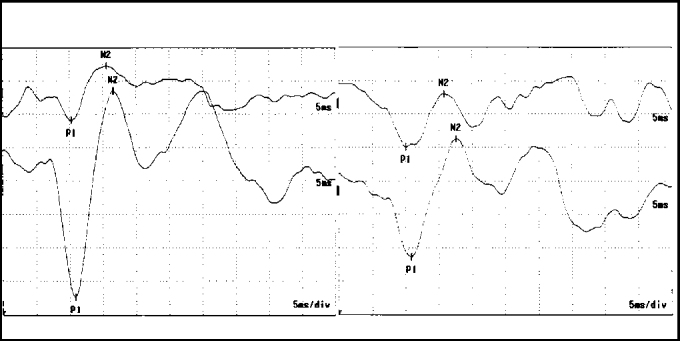
Waves P1 and N2 latency at VEMP in control group subjects. Chart on the right = right ear; chart on the left = left ear.

As to results, we observed that when comparing the right ear with the left ear, the correlation between absence and presence of responses and correlation between absence and presence of symptoms in VEMP did not result in significant difference. Therefore, we could count the ears as isolated normal and not as subjects (Figure 2).

Otoneurological symptoms reported by the patients were extremely varied: hearing loss, tinnitus, ear fullness, imbalance and sudden deafness. Patients referred imbalance as the most frequent symptom. All patients reported these symptoms in some phase of their lives, which could have been sudden, unique, recurrent or progressive.

According to the literature, there are different otoneurological manifestations that can be seen in MS patients. High frequency of imbalance was observed in these cases. They reported complaints of dizziness before and/or after confirmed diagnosis of MS^18^, ^25^, ^26^. They also described some cases of sudden deafness as the first symptom of manifestation of the disease or as a symptom of MS episodes. They also detected that demyelinization could be present in the distal part of the 8th cranial nerve^27^, ^28^, ^29^, ^30^.

**Figure 3:**
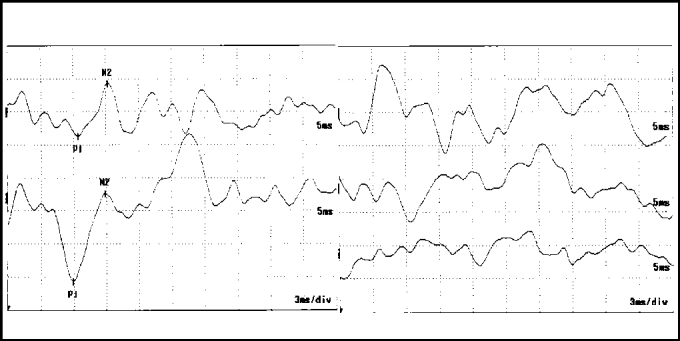
Waves P1 and N2 latency at VEMP in case 1 of the EM group. Absence of response on the right. Chart on the right = right ear; chart on the left = left ear.

In our study, we observed that in the studied group, 30% presented absence of responses (Figure 3). The statistical value obtained (10.59) was considered significant as a result of the critical value^3^, ^9^. The amplitude response P1-N2, wave P1 and wave N2 were higher in patients in the studied group. We also observed a higher amount in symptomatic cases in the studied group than in asymptomatic cases.

The absence of VEMP responses was described in patients that had been submitted to selective section of vestibular nerve^4^, in 71% of the patients with vestibular schwannoma of the affected ear^7^, and in 2 cases of post-neurectomy vestibular schwannoma^13^, ^14^, in 7 cases of vestibular schwannoma (7 out of 28 cases) in which there was affection of inferior vestibular nerve^23^, in 1 case after surgery of cerebellopontine angle tumor^17^, 69% of cerebellopontine angle tumors^31^. Absence of responses and affections of VEMP amplitude^4^, ^16^ could be explained as vestibulospinal tract disorder. Vestibulospinal tract anatomy has been well studied and explored; however, it has not managed to quantify to what extent the vestibulospinal tract has to be affected to cause symptoms and it is also impossible to know the exact location of the affected site. There are other hypotheses to justify the damage: inflammation of superior vestibular nerve, owing to posterior and lateral semicircular canal damage; inflammation of inferior vestibular nerve, which affects the posterior semicircular canal and causes complete inflammation of vestibular ganglion, which includes the superior and inferior vestibular nerve^15^.

Considering that there are few diagnostic tests to assess vestibulospinal tract, VEMP is considered an important method of investigation of this tract.

## CONCLUSION

A study with 15 subjects with MS and 15 subjects in the control group was conducted at IAMSPE and we concluded that:

1. Latency of wave P1 and N2 and amplitude P1-N2 showed increased values in VEMP in patients with MS when compared to the control group.
2. There was no statistically significant difference in results of wave P1 and N2 latency and P1-N2 amplitude in VEMP when comparing both ears.
3. Patients with diagnosis of MS and otoneurological symptoms presented higher frequency of VEMP affection when compared to patients without otoneurological symptoms.
4. VEMP was considered a good diagnostic support method in cases of MS. In addition to being an objective test, it is not invasive and is easy to perform.
